# Serologic Evidence of Various Arboviruses Detected in White-Tailed Deer (*Odocoileus virginianus*) in the United States

**DOI:** 10.4269/ajtmh.17-0180

**Published:** 2017-05-30

**Authors:** Kerri Pedersen, Eryu Wang, Scott C. Weaver, Paul C. Wolf, Adam R. Randall, Kyle R. Van Why, Amelia P.A. Travassos Da Rosa, Thomas Gidlewski

**Affiliations:** 1 U.S. Department of Agriculture, Animal and Plant Health Inspection Service, Wildlife Services, Fort Collins, Colorado;; 2 Institute for Human Infections and Immunity and Department of Microbiology and Immunology, University of Texas Medical Branch, Galveston, Texas;; 3 U.S. Department of Agriculture, Animal and Plant Health Inspection Service, Wildlife Services, Roseburg, Oregon;; 4 U.S. Department of Agriculture, Animal and Plant Health Inspection Service, Wildlife Services, Pittstown, New Jersey;; 5 U.S. Department of Agriculture, Animal and Plant Health Inspection Service, Wildlife Services, Harrisburg, Pennsylvania;; 6 U.S. Department of Agriculture, Animal and Plant Health Inspection Service, Wildlife Services, National Wildlife Research Center, Fort Collins, Colorado

## Abstract

White-tailed deer (*Odocoileus virginianus*) are an abundant mammal with a wide geographic distribution in the United States, which make them good sentinels for monitoring arboviral activity across the country. Exposure to various arboviruses has been detected in white-tailed deer, typically in conjunction with another diagnostic finding. To better assess the exposure of white-tailed deer to seven arboviruses, we tested 1,508 sera collected from 2010 to 2016 for antibodies to eastern equine encephalitis (2.5%), Powassan (4.2%), St. Louis encephalitis, (3.7%), West Nile (6.0%), Maguari (19.4%), La Crosse (30.3%), and bluetongue (7.8%) viruses. At least one arbovirus was detected in 51.3%, and exposure to more than one arbovirus was identified in 17.6% of the white-tailed deer sampled.

## INTRODUCTION

Although a variety of arboviruses have been reported in white-tailed deer (*Odocoileus virginianus*) in the United States, these detections have typically occurred in conjunction with an epizootic or a diagnostic case where the arboviral findings were secondary. Two such arboviruses, West Nile virus (WNV) and St. Louis encephalitis virus (SLEV), are mosquito-borne flaviviruses that although amplified by avian hosts can be transmitted to mammals, including white-tailed deer, but mammals are considered dead-end hosts.^1^ Powassan virus (POWV) is another zoonotic flavivirus, but unlike WNV and SLEV the vector is a tick.^2^ White-tailed deer are the primary host for *Ixodes* spp. ticks, which are responsible for transmitting POWV.^3^ Eastern equine encephalitis virus (EEEV; *Togaviridae*) is an alphavirus that can cause severe neurologic disease in humans, horses (*Equus caballus*), swine (*Sus scrofa*), and birds (*Aves*).^4^ Unlike the other previously described arboviruses, EEEV has been reported to cause mortality in white-tailed deer in some instances.^5,6^ Bluetongue virus (BTV: *Reoviridae*) is an arthropod-borne orbivirus transmitted by *Culicoides* spp. midges that does not affect humans, but can cause hemorrhagic disease in sheep (*Ovis aries*), cattle (*Bos taurus*), and other ruminants. These infections usually occur when naïve animals are introduced into a BTV-endemic area,^7^ or infected vectors are seasonally spread to a new area.^8^ Of the 24 serotypes of BTV circulating worldwide, five (10, 11, 13, 17, and 2) are considered enzootic in the United States,^9^ and all five serotypes except BTV-2 have been associated with mortality in white-tailed deer.^7^ Additional non-enzootic serovars of BTV have been reported in white-tailed deer in the United States. White-tailed deer are not only susceptible to BTV, but may be reservoirs of the virus.^10^ La Crosse virus (LACV; *Bunyaviridae*) is a mosquito-borne orthobunyavirus that causes encephalitis and febrile illness in humans with various signs and symptoms, and it is most commonly reported in the upper Midwest and mid-Atlantic regions of the United States.^8^ Antibodies to LACV have occasionally been reported in white-tailed deer.^11^ Maguari virus (MAGV; *Bunyaviridae*), a subtype of the species Cache Valley virus, is a mosquito-borne orthobunyavirus that has been identified in various parts of South America.^12^ Although antibodies to the virus have been detected in humans, horses, cattle, sheep, water buffalo (*Bubalus bubalis*), and birds, it has only been reported to cause disease in horses.^13,14^

White-tailed deer are the most widespread large mammal in North America.^15^ In areas where they are particularly abundant, they are often targeted by mosquitoes for a blood feeding.^16^ White-tailed deer movement varies by latitude, but even in the northern regions of their range where climate determines movement, they typically do not migrate more than 45 km.^17,18^ This limited movement combined with their wide geographic distribution and abundance make them excellent sentinels for detecting evidence of arbovirus activity within a geographic area.^19^ Although the existence and relative distribution of arboviruses have been well documented in the United States, there are limited opportunities to monitor these pathogens at a national scale. Our objective was to determine whether white-tailed deer across the country are exposed to EEEV, SLEV, WNV, POWV, MAGV, BTV, and LACV, to estimate the seroprevalence of each arbovirus individually, and to determine the frequency of multiple infections.

## MATERIALS AND METHODS

### Sample collection.

The U.S. Department of Agriculture’s Animal and Plant Health Inspection Service, Wildlife Services (WS) remove deer from select areas where populations are not naturally regulated due to lack of predators and hunting restrictions. The purpose of removal is to minimize the problems associated with high densities and overpopulation such as collisions with vehicles or airplanes, herbivorous damage to fruit trees and understory, increased tick abundance, and disease management, in addition to ensuring herd health.^15^ The National Wildlife Disease Program (a branch of WS) utilizes this program to opportunistically collect blood samples from deer, and archive them for future testing. Blood was collected post mortem via intracardiac puncture, and after clotting, the tubes were centrifuged, serum was transferred to cryogenic vials and then stored at −80°C. A standardized datasheet was used to record the date, age, sex, and location (county and GPS coordinates). Deer were classified as fawns (spots on body) or adults. We tested archived sera from 1,508 white-tailed deer from 97 counties in 18 U.S. states and the U.S. Virgin Islands collected between January 2010 and March 2016.

### Serology.

Exposure to EEEV, POWV, SLEV, WNV, and MAGV was determined with a hemagglutination inhibition (HI) assay, which is very useful for serologic screening because it only requires small volumes of serum for testing, and its cross-reactive properties allow for a small number of antigens to detect antibodies against diverse viruses. Plaque reduction neutralization tests (PRNT) are more specific, but this results in a tradeoff between specificity and the ability to screen for immunity to multiple viruses with a limited serum volume. The HI assay was performed in microtiter plates as described previously.^20^ Briefly, four hemagglutination units of mouse brain antigen extracted by sucrose–acetone from each virus were reacted with serially diluted serum starting at a dilution of 1:20. Sera were considered positive if specific antibodies that prevent viruses from binding to goose red blood cells (Lampire Biological Laboratories, Inc., Pipersville, PA) were present. The antigens used were strain North American NJ/60 (EEEV), strain Byers (POWV), strain Parton (SLEV), strain B956 (WNV), and strain BeAr 7272 (MAGV). All virus strains were obtained from the University of Texas Medical Branch World Reference Center for Emerging Viruses and Arboviruses in Galveston, TX. A positive control serum titrated to its endpoint was included for each antigen. Titers ≥ 20 were considered positive, and positive samples were further titrated to their endpoint up to 1:2,560.

Because LACV and BTV do not agglutinate goose red blood cells, exposure was determined using 80% plaque reduction neutralization tests (PRNT_80_) as described previously.^20^ Briefly, serum samples were heat inactivated at 56°C for 30 minutes and then diluted 2-fold in minimal essential medium (MEM, Invitrogen, Carlsbad, CA) supplemented with 2% fetal bovine serum (FBS, Atlanta Biologicals, Atlanta, GA). Then, 100 µL of diluted test serum was mixed with an equal volume of 2% MEM containing approximately 200 plaque-forming units of virus. Next, 100 µL of serum–virus suspension were transferred to a well of confluent Vero cell monolayers in 12-well plates and incubated at 37°C for 60 minutes. For BTV (strain BT8), the cell monolayer was overlaid with 3 mL of a 50:50 mixture of 1% oxoid agar (Fisher Scientific, Pittsburgh, PA) and 2 × MEM (Sigma, St. Louis, MO) containing 4% FBS. The plates were incubated 7 days for plaque formation. For the LACV (strain H 44-71017) assay, an overlay containing 0.4% of agarose was added to each well. The plaques were observed over the next 3 days, and then the viral plaques were counted. For both the BTV and LACV assays, if titers were ≥ 20, the ability of serum samples to neutralize each arbovirus was determined by titrating the sera using serial dilutions (up to 1:640) to confirm a level of antibodies against each virus.

### Data analysis.

Antibody prevalence and 95% confidence intervals were calculated with Microsoft Excel (Microsoft Corp., Redmond, WA).

## RESULTS

Of 1,508 white-tailed deer tested, antibodies were detected for EEEV (2.5%; 95% CI: 1.8–3.4), POWV (4.2%; 95% CI: 3.3–5.3), SLEV (3.7%; 95% CI: 2.8–4.7), WNV (6.0%; 95% CI: 4.9–7.3), MAGV (19.4%; 95% CI: 17.5–21.5), LACV (30.3%; 95% CI: 28.0–32.7), and BTV (7.8%; 95% CI: 6.5–9.2) with prevalence varying widely among states (Table 1). Antibody prevalence for one or more arboviruses was higher in Florida, Indiana, Louisiana, Michigan, Mississippi, and the Virgin Islands than the other states (Table 1).

**Table 1:** Antibody prevalence with 95% CIs of EEEV, POWV, SLEV, WNV, MAGV, LACV, and BTV in white-tailed deer (*Odocoileus virginianus*) by state

State (*n*)	EEEV % (95% CI)	POWV % (95% CI)	SLEV % (95% CI)	WNV % (95% CI)	MAGV % (95% CI)	LACV % (95% CI)	BTV % (95% CI)
Alabama (197)	2.5 (1.1–5.8)	2.5 (1.1–5.8)	2.5 (1.1–5.8)	4.6 (2.4–8.5)	13.7 (9.6–19.2)	35.0 (28.7–41.9)	15.2 (10.9–20.9)
Florida (42)	11.9 (5.2–25.0)	0 (0–8.4)	0 (0–8.4)	0 (0–8.4)	19.1 (10.0–33.3)	45.2 (31.2–60.1)	4.8 (1.3–15.8)
Georgia (88)	1.1 (0.2–6.2)	0 (0–4.2)	0 (0–4.2)	2.3 (0.6–7.9)	19.3 (12.4–28.8)	11.4 (6.3–19.7)	4.6 (1.8–11.1)
Illinois (43)	0 (0–8.2)	4.7 (1.3–15.5)	4.7 (1.3–15.5)	2.3 (0.4–12.1)	25.6 (14.9–40.2)	11.6 (5.1–24.5)	4.7 (1.3–15.5)
Indiana (45)	2.2 (0.4–11.6)	20.0 (10.9–33.8)	15.6 (7.8–28.8)	13.3 (6.3–26.2)	28.9 (17.7–43.4)	28.9 (17.7–43.4)	0 (0–7.9)
Kentucky (8)	0 (0–32.4)	0 (0–32.4)	0 (0–32.4)	0 (0–32.4)	25.0 (7.2–59.1)	0 (0–32.4)	0 (0–32.4)
Louisiana (75)	4.0 (1.4–11.1)	5.3 (2.1–12.9)	13.3 (7.4–22.8)	18.7 (11.5–28.9)	29.3 (20.2–40.4)	52.0 (40.9–62.9)	20.0 (12.5–30.4)
Maine (3)	0 (0–56.2)	0 (0–56.2)	0 (0–56.2)	0 (0–56.2)	0 (0–56.2)	66.7 (20.8–93.9)	0 (0–56.2)
Michigan (227)	1.3 (0.5–3.8)	0 (0–1.7)	2.2 (0.9–5.1)	2.2 (0.9–5.1)	16.7 (12.5–22.1)	37.4 (31.4–43.9)	4.0 (2.1–7.4)
Minnesota (237)	1.7 (0.7–4.3)	3.8 (2.0–7.1)	4.2 (2.3–7.6)	8.9 (5.9–13.2)	16.0 (11.9–21.2)	29.1 (23.7–35.2)	3.0 (1.4–6.0)
Mississippi (32)	3.1 (0.6–15.7)	0 (0–10.7)	3.1 (0.6–15.7)	3.1 (0.6–15.7)	9.4 (3.2–24.2)	59.4 (42.3–74.5)	43.8 (28.2–60.7)
North Carolina (3)	0 (0–56.2)	0 (0–56.2)	0 (0–56.2)	0 (0–56.2)	0 (0–56.2)	0 (0–56.2)	0 (0–56.2)
New Jersey (178)	3.4 (1.6–7.2)	9.6 (6.1–14.8)	3.9 (1.9–7.9)	9.0 (5.6–14.1)	11.2 (7.4–16.7)	26.4 (20.5–33.3)	3.9 (1.9–7.9)
New York (2)	0 (0–65.8)	0 (0–65.8)	0 (0–65.8)	0 (0–65.8)	0 (0–65.8)	0 (0–65.8)	0 (0–65.8)
Ohio (21)	0 (0–15.5)	0 (0–15.5)	0 (0–15.5)	0 (0–15.5)	19.1 (7.7–40.0)	14.3 (5.0–34.6)	4.8 (0.9–22.7)
Pennsylvania (278)	2.9 (1.5–5.6)	6.1 (3.9–9.6)	2.9 (1.5–5.6)	4.3 (2.5–7.4)	21.2 (16.8–26.4)	26.6 (21.8–32.1)	4.7 (2.8–7.8)
Virgin Islands (18)	0 (0–17.6)	0 (0–17.6)	0 (0–17.6)	16.7 (5.8–39.2)	0 (0–17.6)	0 (0–17.6)	72.2 (49.1–87.5)
Virginia (10)	0 (0–27.8)	0 (0–27.8)	0 (0–27.8)	0 (0–27.8)	30.0 (10.8–60.3)	30.0 (10.8–60.3)	10.0 (1.8–40.4)
Wyoming (1)	0 (0–79.4)	0 (0–79.4)	0 (0–79.4)	0 (0–79.4)	0 (0–79.4)	0 (0–79.4)	0 (0–79.4)

BTV = bluetongue virus; CI = confidence interval; EEEV = eastern equine encephalitis virus; LACV = La Crosse virus; MAGV = Maguari virus; POWV = Powassan virus; SLEV = St. Louis encephalitis virus; WNV = West Nile virus.

Age classes were comprised primarily of adults (75.7%) followed by fawns (23.8%) and unknown age (not recorded; 0.4%). Adults were more likely than fawns to be antibody positive for MAGV, LACV, and BTV, but there were no differences between age classes for the other arboviruses (Table 2). The majority of the deer sampled were females (63.2%), followed by males (36.0%) and unknown (not recorded; 0.7%). There were no apparent associations between antibody prevalence and sex except that females were more likely than males to be antibody positive for MAGV and LACV (Table 3).

**Table 2:** Antibody prevalence with 95% CIs of white-tailed deer (*Odocoileus virginianus*) by age class collected from 2010 to 2016, and tested for exposure to EEEV, POWV, SLEV, WNV, MAGV, LACV, and BTV with HI and PRNT_80_

Age class (*n*)			
Arbovirus (test)	Fawns (359) % (95% CI)	Adults (1142) % (95% CI)	Unknown (7) % (95% CI)
EEEV (HI)	2.0 (1.0–4.0)	2.6 (1.9–3.7)	0 (0–35.4)
POWV (HI)	4.2 (2.6–6.8)	4.2 (3.2–5.5)	0 (0–35.4)
SLEV (HI)	3.1 (1.7–5.4)	3.9 (2.9–5.2)	0 (0–35.4)
WNV (HI)	5.0 (3.2–7.8)	6.2 (5.0–7.8)	14.3 (2.6–51.3)
MAGV (HI)	8.1 (5.7–11.4)	22.8 (20.4–25.3)	57.1 (25.1–84.2)
LACV (PRNT_80_)	17.3 (13.7–21.5)	34.4 (31.7–37.2)	28.6 (8.2–64.1)
BTV (PRNT_80_)	3.6 (2.1–6.1)	8.9 (7.4–10.7)	28.6 (8.2–64.1)

BTV = bluetongue virus; CI = confidence interval; EEEV = eastern equine encephalitis virus; HI = hemagglutination inhibition; LACV = La Crosse virus; MAGV = Maguari virus; POWV = Powassan virus; PRNT_80_ = 80% plaque reduction neutralization assay; SLEV = St. Louis encephalitis virus; WNV = West Nile virus.

**Table 3:** Antibody prevalence with 95% CIs of white-tailed deer (*Odocoileus virginianus*) by sex collected from 2010 to 2016, and tested for exposure to EEEV, POWV, SLEV, WNV, MAGV, LACV, and BTV with HI and PRNT_80_

Sex			
Arbovirus	Male (542) % (95% CI)	Female (955) % (95% CI)	Unknown (11) % (95% CI)
EEEV	2.8 (1.7–4.5)	2.3 (1.5–3.5)	0 (0–25.9)
POWV	5.0 (3.5–7.2)	3.8 (2.7–5.2)	0 (0–25.9)
SLEV	3.9 (2.6–5.9)	3.6 (2.6–4.9)	0 (0–25.9)
WNV	5.4 (3.8–7.6)	6.4 (5.0–8.1)	0 (0–25.9)
MAGV	13.5 (10.9–16.6)	22.7 (20.2–25.5)	27.3 (9.8–56.6)
LACV	24.7 (21.3–28.5)	33.4 (30.5–36.5)	36.4 (15.2–64.6)
BTV	6.8 (5.0–9.3)	8.4 (6.8–10.3)	0 (0–25.9)

BTV = bluetongue virus; CI = confidence interval; EEEV = eastern equine encephalitis virus; HI = hemagglutination inhibition; LACV = La Crosse virus; MAGV = Maguari virus; POWV = Powassan virus; PRNT_80_ = 80% plaque reduction neutralization assay; SLEV = St. Louis encephalitis virus; WNV = West Nile virus.

In 51.3% of the samples, antibodies to at least one of the arboviruses were detected. At least two prior infections were detected in 17.6% of the deer, and the most common combination was MAGV and LACV (*N* = 97), followed by LACV and BTV (*N* = 29), and MAGV, LACV, and BTV (*N* = 11). These double seropositives are unlikely to represent cross-reactions because they represent different bunyavirus serogroups or different virus families. All other combinations occurred in less than 10 animals. There were no differences in antibody prevalence among seasons (Table 4).

**Table 4:** Antibody prevalence with 95% CIs of white-tailed deer (*Odocoileus virginianus*) by season collected from 2010 to 2016, and tested for exposure to EEEV, POWV, SLEV, WNV, MAGV, LACV, and BTV with HI and PRNT_80_

Season (*n*)				
Arbovirus	Fall (351) % (95% CI)	Spring (390) % (95% CI)	Summer (149) % (95% CI)	Winter (618) % (95% CI)
EEEV	2.9 (1.6–5.2)	3.6 (2.2–5.9)	1.3 (0.4–4.8)	1.8 (1.0–3.2)
POWV	5.4 (3.5–8.3)	2.1 (1.0–4.0)	0.7 (0.1–3.7)	5.7 (4.1–7.8)
SLEV	4.3 (2.6–6.9)	2.8 (1.6–5.0)	2.0 (0.7–5.8)	4.2 (2.9–6.1)
WNV	7.7 (5.3–11.0)	3.6 (2.2–5.9)	1.3 (0.4–4.8)	7.6 (5.8–10.0)
MAGV	23.7 (19.5–28.4)	16.2 (12.8–20.1)	31.5 (24.6–39.4)	16.2 (13.5–19.3)
LACV	34.2 (29.4–39.3)	33.3 (28.8–38.2)	48.3 (40.4–56.3)	21.8 (18.8–25.3)
BTV	10.8 (8.0–14.5)	11.0 (8.3–14.5)	5.4 (2.8–10.2)	4.5 (3.2–6.5)

BTV = bluetongue virus; CI = confidence interval; EEEV = eastern equine encephalitis; HI = hemagglutination inhibition; LACV = La Crosse virus; MAGV = Maguari virus; POWV = Powassan virus; PRNT_80_ = 80% plaque reduction neutralization assay; SLEV = St. Louis encephalitis; WNV = West Nile virus.

## DISCUSSION

It is not surprising antibody prevalence to MAGV, LACV, and BTV was higher in adults than fawns because adults have more opportunities for exposure over time. This pattern has been documented repeatedly with arthropod-borne diseases in other wildlife systems^21,22^; however, adults were not more likely to be exposed to EEEV, POWV, SLEV, or WNV. Other variables also influence exposure patterns in adults and fawns including variations in latitude or season that the deer was exposed, length of maternal antibody persistence, and distribution of the mosquito vector.^23^ There were no differences in antibody prevalence between males and females for any of the arboviruses with the exception of MAGV and LACV. Antibody prevalence of both of these arboviruses was higher in females. The mechanisms behind the differences in sexes documented here are unknown, but foundational work in other wildlife disease systems have implicated hormonal differences between males and females causing altered immune response and behavioral variations between the sexes that affect the likelihood of exposure.^24,25^

Although it does not appear that most arboviruses cause disease in white-tailed deer, EEEV has been identified as the cause of neurologic disease in the species.^5,6^ It is also interesting to note that the antibody prevalence we detected for EEEV (2.5%) was lower than that for any of the other arboviruses. If EEEV infection is often fatal in deer, this could be reflected in low seroprevalence based on few survivors. However, a more likely explanation is that the enzootic vector mosquito in most parts of North America, *Culiseta melanura*, is highly ornithophilic.^26^ Deer are considered an excellent indicator for POWV activity.^27^ Since we detected POWV in seven (Alabama, Illinois, Indiana, Louisiana, Minnesota, New Jersey, and Pennsylvania) of the 19 states sampled, it appears that either many human cases are underreported or deer are more likely to become infected due to higher rates of exposure to the tick vector.

Though there appeared to be higher antibody prevalence in some of the states for certain arboviruses (Table 1), no definitive associations could be made because there was wide variation in sample sizes among states and not all states were represented. However, it is interesting to note that all of the WNV antibody-positive deer in Indiana were collected in Porter county in 2013 (*N* = 6), which also reported human cases in 2010 (*N* = 2) and 2012–2014 (*N* = 4, 3, 3, respectively). Also, in Louisiana, all of the WNV antibody-positive samples (*N* = 14) were collected in 2012, which was a peak year for reported human cases (225 in 2012 compared with 54, 125, and 51 in 2013, 2014, and 2015, respectively (https://diseasemaps.usgs.gov/mapviewer/).

Although MAGV was not detected north of Trinidad a few years ago,^28^ this arbovirus may now be common in the United States since we detected antibodies in 19.4% of the white-tailed deer that we tested. However, we cannot rule out that some of these seropositives represent cross-reactions with the close relative Cache Valley virus; unfortunately sample volumes were not adequate to test against the latter. Further studies to investigate whether other species have been exposed to MAGV and to redefine the geographic distribution are recommended since it appears to exist at latitudes north of where detected previously. MAGV has been identified in other countries without causing an epidemic,^29^ suggesting that it may have been in the United States but not detected, since no active surveillance was occurring for the pathogen.

Although various studies have been conducted across the United States documenting evidence of the arboviruses we report here, we believe this to be the most comprehensive survey of white-tailed deer because of the geographic extent of the samples, as well as the number of deer and combination of arboviruses examined. Though our results do not suggest that white-tailed deer serve as amplifying hosts for the viruses we studied,^1,19^ surveys of white-tailed deer are useful for identifying localized foci of arboviral activity as has been suggested previously.^30^ They can also provide a rapid, cost-effective way of monitoring arboviral activity in states since human cases tend to be biased toward those with symptoms and identifying the location of exposure is difficult.^30^ Surveys of this type may become even more important as human modifications of the environment result in secondary effects on the abundance and distribution of vectors and vertebrates that are intermediate hosts.^31^

## References

[1] Farajollahi A Gates R Crans W Komar N, 2004 Serologic evidence of West Nile virus and St. Louis encephalitis virus infections in white-tailed deer (*Odocoileus virginianus*) from New Jersey, 2001. Vector-Borne Zoonotic Dis 4: 379–383. 15671740 10.1089/vbz.2004.4.379

[2] Hardy JL, 1994 Arboviral zoonoses of North America. Beran GW, ed. *Handbook of Zoonoses Section B: Viral*. Boca Raton, FL: CRC Press LLC, 185–200.

[3] Nofchissey RA, et al., 2013 Seroprevalence of Powassan virus in New England deer, 1979–2010. Am J Trop Med Hyg 88: 1159–1162. 23568288 10.4269/ajtmh.12-0586 PMC3752817

[4] Scott TW Weaver SC, 1989 Eastern equine encephalomyelitis virus: epidemiology and evolution of mosquito transmission. Adv Virus Res 37: 277–328. 2574935 10.1016/s0065-3527(08)60838-6

[5] Tate CM Howerth EW Stallknecht DE Allison AB Fischer JR Mead DG, 2005 Eastern equine encephalitis in a free-ranging white-tailed deer (*Odocoileus virginianus*). J Wildl Dis 41: 241–245. 15827230 10.7589/0090-3558-41.1.241

[6] Schmitt SM Cooley TM Fitzgerald SD Bolin SR Lim A Schaefer SM Kiupel M Maes RK Hogle SA O’Brien DJ, 2007 An outbreak of eastern equine encephalitis virus in free-ranging white-tailed deer in Michigan. J Wildl Dis 43: 635–644. 17984258 10.7589/0090-3558-43.4.635

[7] Stallknecht D Howerth E, 2004 Epidemiology of bluetongue and epizootic haemorrhagic disease in wildlife: surveillance methods. Vet Ital 40: 203–207. 20419663

[8] Eldridge BF Scott TW Day JF Tabachnick WJ, 2004 Arbovirus diseases. Eldridge BF, Edman JD, eds. *Medical Entomology: A Textbook on Public Health and Veterinary Problems Caused by Arthropods*, revised edition. New York, NY: Springer Science and Business Media, B.V., 415–460.

[9] Ruder MG Lysyk TJ Stallknecht DE Foil LD Johnson DJ Chase CC Dargatz DA Gibbs EPJ, 2015 Transmission and epidemiology of bluetongue and epizootic hemorrhagic disease in North America: current perspectives, research gaps, and future directions. Vector-Borne Zoonot 15: 348–363. 10.1089/vbz.2014.1703 26086556

[10] Johnson DJ Ostlund EN Stallknecht DE Goekjian VH Jenkins-Moore M Harris SC, 2006 First report of bluetongue virus serotype 1 isolated from a white-tailed deer in the United States. J Vet Diagn Invest 18: 398–401. 16921883 10.1177/104063870601800415

[11] Nagayama J Komar N Levine J Biggerstaff B Apperson C, 2001 Bunyavirus infections in North Carolina white-tailed deer (*Odocoileus virginianus*). Vector-Borne Zoonot 1: 169–171. 10.1089/153036601316977778 12678047

[12] Calisher CH Francy DB Smith GC Muth DJ Lazuick JS Karabatsos N Jakob W McLean RG, 1986 Distribution of Bunyamwera serogroup viruses in North America, 1956–1984. Am J Trop Med Hyg 35: 429–443. 2869708 10.4269/ajtmh.1986.35.429

[13] Swanepoel R, 2000 Bunyaviridae. Zuckerman AJ, Banatvala JE, Pattison JR, eds. *Principles and Practice of Clinical Virology*. New York, NY: John Wiley and Sons Ltd., 515–549.

[14] Monath T Sabattini M Pauli R Daffner J Mitchell C Bowen G Cropp C, 1985 Arbovirus investigations in Argentina, 1977–1980. IV. Serologic surveys and sentinel equine program. Am J Trop Med Hyg 34: 966–975. 2863991

[15] DeNicola AJ VerCauteren KC Curtis PD Hyngstrom SE, 2000 *Managing White-Tailed Deer in Suburban Environments. A Technical Guide*. Ithaca, NY: Cornell Cooperative Extension, the Wildlife Society’s Wildlife Damage Management Working Group, and the Northeast Wildlife Damage Research and Outreach Cooperative, 52.

[16] Apperson CS, 2004 Host feeding patterns of established and potential mosquito vectors of West Nile virus in the eastern United States. Vector-Borne Zoonot 4: 71–82. 10.1089/153036604773083013 PMC2581457 15018775

[17] Nelson ME Mech LD Frame PF, 2004 Tracking of white-tailed deer migration by global positioning system. J Mammal 85: 505–510.

[18] Sabine DL Morrison SF Whitlaw HA Ballard WB Forbes GJ Bowman J, 2002 Migration behavior of white-tailed deer under varying winter climate regimes in New Brunswick. J Wildl Manage 66: 718–728.

[19] Emmons RW, 1968 Serologic survey of a deer herd in California for arbovirus infections. Wildl Dis 4: 78–80. 5691432 10.7589/0090-3558-4.3.78

[20] Beaty BJ Calisher CH Shope RE, 1995 Arboviruses. Lennette EH, Lenette DA, Lennette ET, eds. *Diagnostic Procedures for Viral, Rickettsial, and Chlamydial Infections*. Washington, DC: American Public Health Association, 189–212.

[21] Neitzel DF Grimstad PR, 1991 Serological evidence of California group and Cache Valley virus infection in Minnesota white-tailed deer. J Wildl Dis 27: 230–237. 1906113 10.7589/0090-3558-27.2.230

[22] García I Napp S Casal J Perea A Allepuz A Alba A Carbonero A Arenas A, 2009 Bluetongue epidemiology in wild ruminants from southern Spain. Eur J Wildl Res 55: 173–178.

[23] Calisher CH, 1994 Medically important arboviruses of the United States and Canada. Clin Microbiol Rev 7: 89–116. 8118792 10.1128/cmr.7.1.89 PMC358307

[24] Klein SL, 2003 Parasite manipulation of the proximate mechanisms that mediate social behavior in vertebrates. Physiol Behav 79: 441–449. 12954438 10.1016/s0031-9384(03)00163-x

[25] Plowright RK Field HE Smith C Divljan A Palmer C Tabor G Daszak P Foley JE, 2008 Reproduction and nutritional stress are risk factors for Hendra virus infection in little red flying foxes (*Pteropus scapulatus*). *Proc R Soc Lond B: [Biol]* 275: *861–869*. 10.1098/rspb.2007.1260 PMC2596896 18198149

[26] Molaei G Andreadis TG, 2006 Identification of avian- and mammalian-derived bloodmeals in *Aedes vexans* and *Culiseta melanura* (Diptera: Culicidae) and its implication for West Nile virus transmission in Connecticut, U.S.A. J Med Entomol 43: 1088–1093. 17017250 10.1603/0022-2585(2006)43[1088:IOAAMB]2.0.CO;2

[27] Whitney E Roz AP Rayner GA Deibel R, 1969 Serologic survey for arbovirus activity in deer sera from nine counties in New York State. Wildl Dis 5: 392–397. 5388983 10.7589/0090-3558-5.4.392

[28] Calisher CH Lazuick JS Lieb S Monath TP Castro KG, 1988 Human infections with Tensaw virus in south Florida: evidence that Tensaw virus subtypes stimulate the production of antibodies reactive with closely related Bunyamwera serogroup viruses. Am J Trop Med Hyg 39: 117–122. 2899978 10.4269/ajtmh.1988.39.117

[29] Iversson LB Silva RA Rosa A Barros VLR, 1993 Circulation of eastern equine encephalitis, western equine encephalitis, Ilheus, Maguari and Tacaiuma viruses in equines of the Brazilian Pantanal, South America. Rev Inst Med Trop Sao Paulo 35: 355–359. 8115796 10.1590/s0036-46651993000400009

[30] Santaella J McLean R Hall JS Gill JS Bowen RA Hadow HH Clark L, 2005 West Nile virus serosurveillance in Iowa white-tailed deer (1999–2003). Am J Trop Med Hyg 73: 1038–1042. 16354809

[31] Tsai TF, 1994 The RNA arboviral zoonoses. Beran GW, ed. *Handbook of Zoonoses Section B: Viral*. Boca Raton, FL: CRC Press LLC, 4–5.

